# Automatic deep learning-based consolidation/collapse classification in lung ultrasound images for COVID-19 induced pneumonia

**DOI:** 10.1038/s41598-022-22196-y

**Published:** 2022-10-20

**Authors:** Nabeel Durrani, Damjan Vukovic, Jeroen van der Burgt, Maria Antico, Ruud J. G. van Sloun, David Canty, Marian Steffens, Andrew Wang, Alistair Royse, Colin Royse, Kavi Haji, Jason Dowling, Girija Chetty, Davide Fontanarosa

**Affiliations:** 1 School of Clinical Sciences, Queensland University of Technology, Gardens Point Campus, 2 George St, Brisbane, QLD 4000 Australia; 2 Faculty of Engineering, Queensland University of Technology, Gardens Point Campus, 2 George St, Brisbane, QLD 4000 Australia; 3 Centre for Biomedical Technologies (CBT), Queensland University of Technology, Brisbane, QLD 4000 Australia; 4 School of IT & Systems, Faculty of Science and Technology, University of Canberra, 11 Kirinari Street, Bruce, ACT 2617 Australia; 5 Department of Surgery (Royal Melbourne Hospital), University of Melbourne, Royal Parade, Parkville, VIC 3050 Australia; 6 Outcomes Research Consortium, Cleveland Clinic, Cleveland, OH USA; 7 CSIRO Health and Biosecurity, The Australian eHealth Research Centre, Herston, QLD 4029 Australia; 8 Department of Electrical Engineering, Eindhoven University of Technology, 5600 MB Eindhoven, The Netherlands; 9 Department of Medicine and Nursing, Monash University, Wellington Road, Clayton, VIC 3800 Australia

**Keywords:** Machine learning, Medical imaging

## Abstract

Our automated deep learning-based approach identifies consolidation/collapse in LUS images to aid in the identification of late stages of COVID-19 induced pneumonia, where consolidation/collapse is one of the possible associated pathologies. A common challenge in training such models is that annotating each frame of an ultrasound video requires high labelling effort. This effort in practice becomes prohibitive for large ultrasound datasets. To understand the impact of various degrees of labelling precision, we compare labelling strategies to train fully supervised models (frame-based method, higher labelling effort) and inaccurately supervised models (video-based methods, lower labelling effort), both of which yield binary predictions for LUS videos on a frame-by-frame level. We moreover introduce a novel sampled quaternary method which randomly samples only 10% of the LUS video frames and subsequently assigns (ordinal) categorical labels to all frames in the video based on the fraction of positively annotated samples. This method outperformed the inaccurately supervised video-based method and more surprisingly, the supervised frame-based approach with respect to metrics such as precision-recall area under curve (PR-AUC) and F1 score, despite being a form of inaccurate learning. We argue that our video-based method is more robust with respect to label noise and mitigates overfitting in a manner similar to label smoothing. The algorithm was trained using a ten-fold cross validation, which resulted in a PR-AUC score of 73% and an accuracy of 89%. While the efficacy of our classifier using the sampled quaternary method significantly lowers the labelling effort, it must be verified on a larger consolidation/collapse dataset, our proposed classifier using the sampled quaternary video-based method is clinically comparable with trained experts’ performance.

## Introduction

Lung ultrasound (LUS) imaging has been used to identify and monitor lung changes associated with the highly contagious respiratory infections resulting from COVID-19^1^. Ultrasound has proven to be more sensitive and specific than X-ray imaging and to be on par with computed tomography (CT)^2^ in detecting imaging patterns associated with lung pathologies. Being a portable, safe non-ionising imaging modality, Ultrasound is ideal for bedside, point-of-care examinations, such that is required for isolated contagious COVID-19 patients. These patients require periodic assessment and imaging of identified lung changes, which assists in monitoring the severity of the COVID-19 respiratory disease progression.

COVID-19 respiratory infection can present with a myriad of lung pathologies and resultant ultrasound imaging patterns, as the disease progresses from acute through to chronic stages. In the initial stages of infection, associated lung changes that may be appreciated on ultrasound include irregular pleura, thickened pleura, multiple B-line artifacts indicative of interstitial syndrome, and collapse. As the infection progresses, more pronounced pleural irregularities, tissue like echotexture suggestive of consolidation and scarring may develop^3,4^. While low in incidence, other respiratory pathologies including pneumothorax^5^ and pleural effusion^6^ remain relevant for exclusion. As the COVID-19 respiratory infection persists, LUS may be used to assist with clinical response to intensive therapies such as prone ventilation or high positive end-expiratory pressure^7^.

The workflow for COVID-19 LUS assessment typically consists of a highly trained and experienced LUS operator acquiring the images using a well-outlined protocol (for example the one described in^8^) and interpretation of these images for any visible ultrasound imaging pattern changes associated with lung pathology. This requires considerable time and resources considering the extensive training and experience required^9^ to perform, acquire and interpret the LUS images. These hindrances have been compounded during the COVID-19 pandemic, where the directly supervised training of additional operators to perform complex ultrasounds, with frequent patient contact, has been prohibitive. These challenges may be alleviated by using machine learning-based automatic interpretation in real-time (at the patient’s bedside) to facilitate the training and clinical use of lung ultrasound.

### Related works

The related literature on automatic identification of COVID-19 induced lung pathologies utilising LUS imaging^10^ involves applying Deep Learning (DL) algorithms trained on COVID-19 LUS images^11,11–15^ and on identifying imaging patterns such as B-lines and pleural thickening that are associated with COVID-19^1,16–20^ with inter- and intra-observer studies^21,22^. These DL algorithms consist of convolutional neural networks (CNNs), which are presently considered the state-of-the -art for automated image analysis given their capability to extract low and high-level image features automatically. La Salvia M et al.^1^ implemented a 4-class classification approach, where class 0 was associated with the identification of A lines, class 1 referred to the presence of the shape of pleural lining, class 2 to broken or damaged pleural lines with the addition of consolidated areas, and finally class 3 to the appearance of tissue-like patterns with/without consolidation. This approach was then extended to a 7 classes classification problem, with the addition of 3 classes including the presence of B-lines or different degrees of image artefacts. In contrast, Baloescu C et al.^17^ created a custom supervised CNN to automatically detect B-lines in non-COVID patients and compared their algorithm results to well-known algorithms such as ResNet^23^ and DenseNet^24^.

Alternatively, Ebadi et al^16^ use a video-based Kinetics-I3D deep learning network that can identify automatically alveolar consolidation and/or pleural effusion alongside A and B lines in LUS videos.

Other works include Roy S et al.^14^ who developed a COVID-19 severity scoring algorithm trained on a LUS dataset of patients. This dataset consisted of patients with mild (label = 1) to severe (label = 3) COVID-19 associated imaging patterns which was validated in a frame and video method by trained sonographers. These images and their associated labels were used to train a DL algorithm in a weakly supervised way by providing segmented and image-based annotated ground truth labels to a Spatial Transform Network (STN) to automatically localise and classify severity of COVID-19 on a frame-by-frame basis. A class was assigned to each severity stage by providing a segmented ground truth label and was fed into a STN that determined the spatial relationship between the imaging patterns associated with that pathology and its location in each video and associated frame. Finally, our group in a previous work^25^ focused on automatic identification and classification of pleural effusion using a modified DL COVID severity algorithm implemented initially by^14^.

The approach proposed here further develops the pathology classification algorithm of^25^ by identifying consolidation/collapse in patients that have respiratory imaging patterns that may be associated with late-stage COVID-19 infection. The novelty of this work includes the application to a unique consolidation/collapse dataset and the development and testing of several different labelling strategies, including a novel efficient data labelling method.

This development of the video-based method is based on the sampling of frames from LUS videos and bears similarity to label smoothing^26^, a method that often improves the performance of classifiers trained on noisy labels (i.e. labels that may be incorrect)^27^. The opposing points of view for label smoothing are that (1) uniform noise is being injected to the labels hence accentuating the problem of noisy labels and that (2) the blurring of label noise may reduce the soft label/class in any one training example^27^. In practice, however, label smoothing has been demonstrated to be effective at improving classifier performance^27^.

## Materials and methods

### Dataset

**Figure 1:**
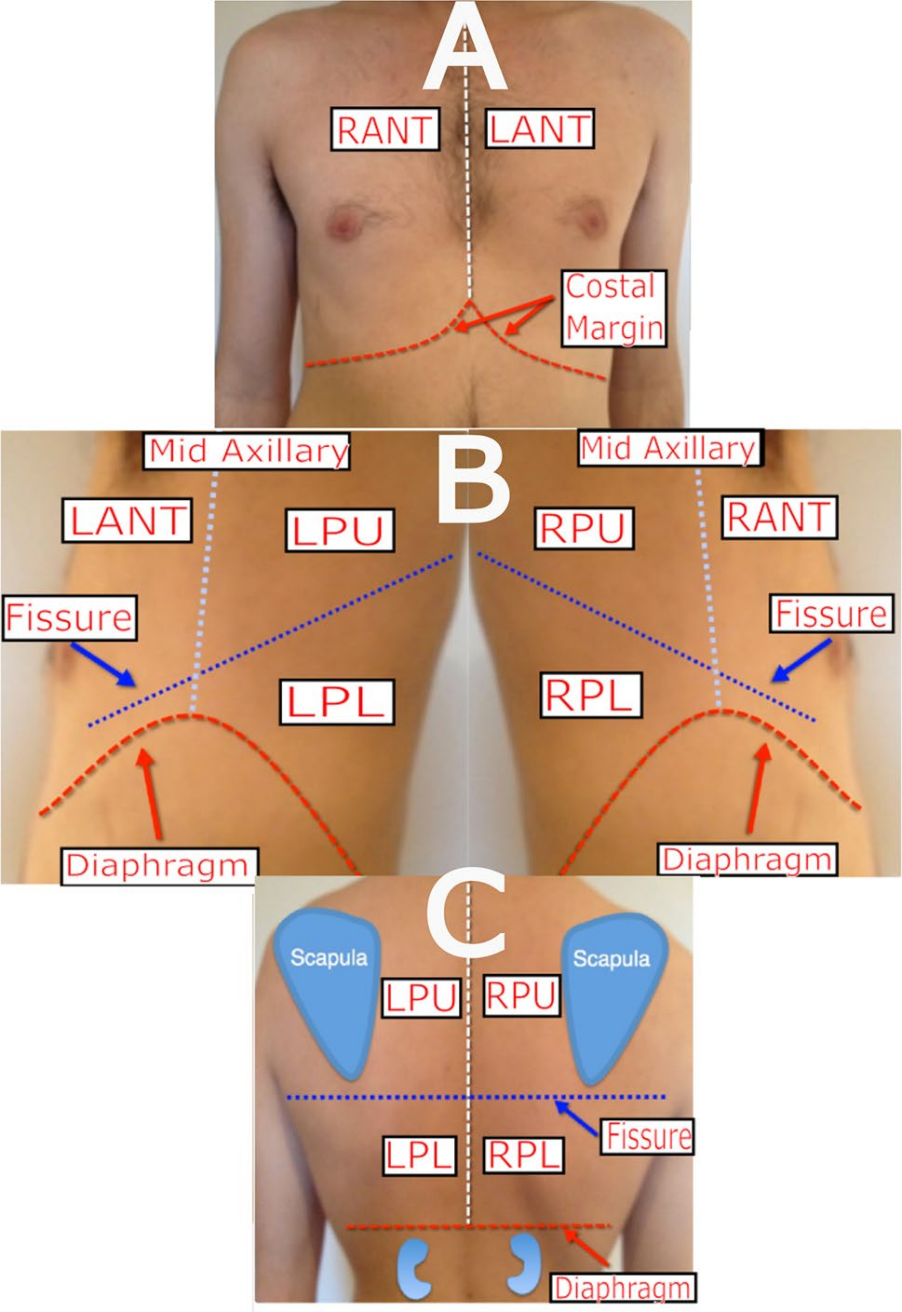
Describes the scanning locations: (**A**) Right Anterior (RANT) and Left Anterior (LANT); (**B**) Left Posterior Upper (LPU), Left Posterior Lower (LPL), Right Posterior Upper (RPU), and Right Posterior Lower (RPL); (**C**) Posterior view for LPU, RPU, RPL and LPL *Source: Adapted from *^30^.

**Figure 2:**
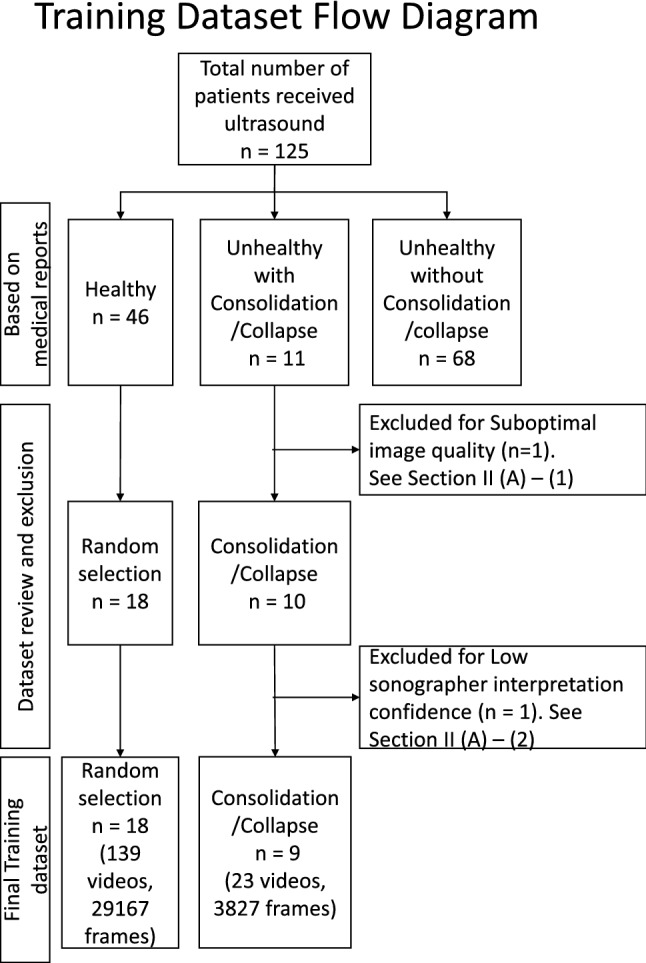
Distribution of the patients before any processing (original dataset) to the final training dataset (after processing, data exclusion, and clinical exclusion).

This study was approved by The Melbourne Health Human Research Ethics Committee (HREC/66935/MH-2020) and was performed in accordance with the Declaration of Helsinki. Lung ultrasound images used in this study were acquired from a previous study where written informed consent was obtained from all participating patients^28^ (Melbourne Health Human Research Ethics Committee approval HREC/18/MH/269, trial registration: http://www.ANZCTR.org.au/ACTRN12618001442291.aspx). All patients were admitted to the Royal Melbourne Hospital under an Internal Medicine unit with a cardiorespiratory-related presentation. Using a Sonosite X-Porte portable ultrasound imaging system (Fujifilm, Bothell, WA, USA) with a 1-5 MHz phased array transducer in Cardiac preset ( acoustic working frequency: 1.72 MHz, mechanical index: 1.3, soft-tissue thermal index: 0.9, focal optimization: Gen, default penetration depth: 15 cm, pulse repetition rate: 2933 Hz, scan repetition rate: 34.1 Hz, tissue harmonic imaging: On), the lung ultrasound examination was performed by an experienced physician trained in point-of-care ultrasound (XC)^28^. The examination followed a standardized iLungScan protocol (The University of Melbourne, Ultrasound Education Group^29^). Patients were positioned in a supine position. Six distinct lung scanning zones were examined at least once (Figs. 1, 2). Images were reviewed for diagnostic accuracy and quality assurance by one of the lung ultrasound experts (DC, AR or CR).

**Figure 3:**
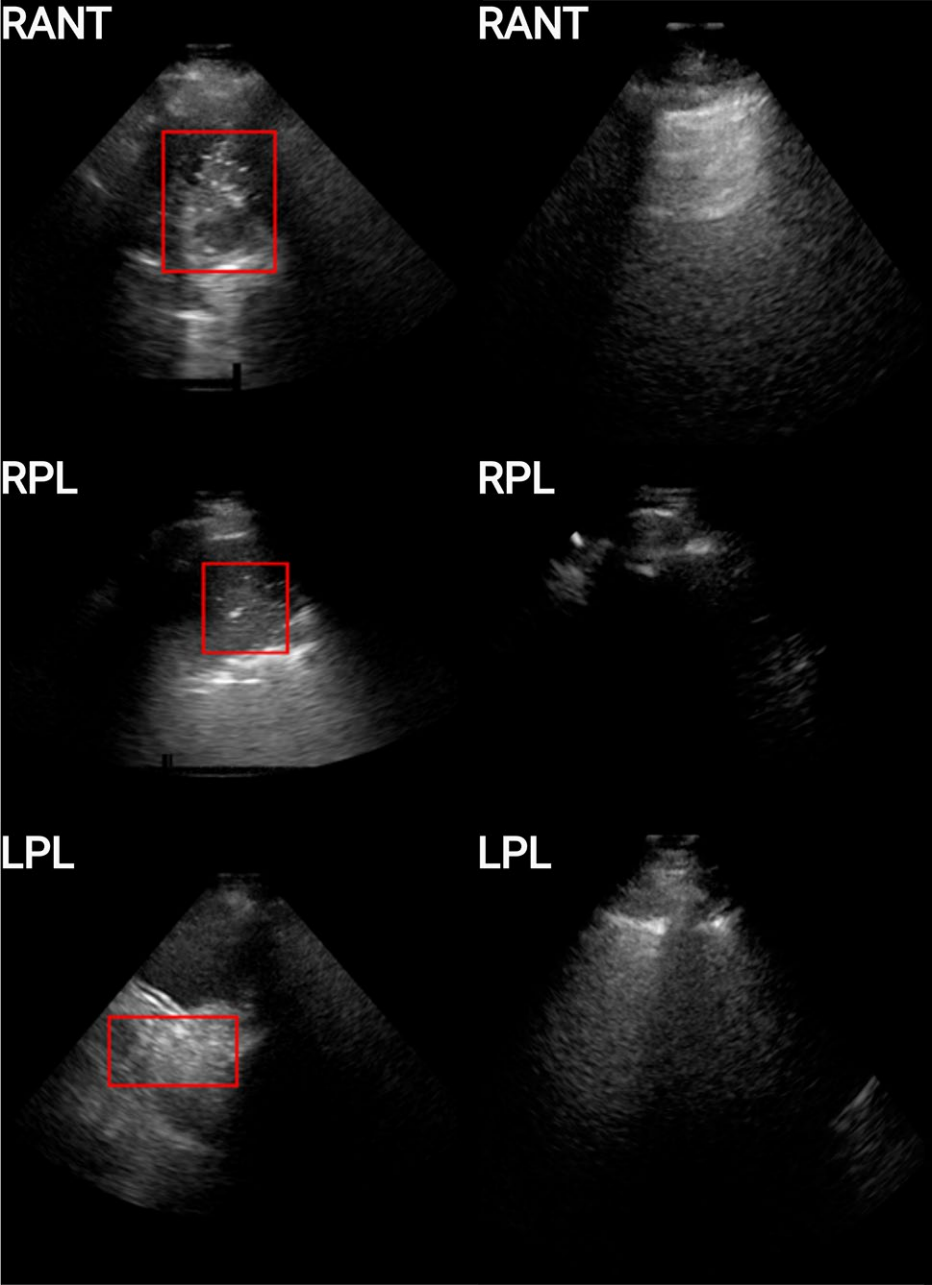
Examples of unhealthy (left column) and healthy (right column) patients for 3 scanning regions (viz. RANT, RPL, LPL). The unhealthy patients are those for which consolidation is present, and are depicted here with a red bounding box encompassing the imaging patterns associated with the pathology, while the healthy patients are those for which no imaging patterns associated with consolidation/collapse or other pathologies.

The original LUS image dataset consisted of 125 patients, it included a range of imaging patterns associated with multiple pathologies. Images were stored in DICOM format. The performing physician recorded the image interpretation on a standardized report form. Based on the available medical reports, we identified 46 patients with lungs of healthy appearance (healthy patients), and 11 patients with lungs of unhealthy appearance consisting of evidence of consolidation/collapse (unhealthy patients). These 11 unhealthy (consolidation/collapse) patients’ LUS images were screened by two independent experts in ultrasound (AW and MS) for image quality (Section 2: A1) and interpretation confidence (Section 2: A1). This resulted in the selection of 9 unhealthy consolidation/collapse patients. Eighteen healthy patients out of the 46 were then randomly selected for this study. A normal lung pattern was identified by the presence of lung sliding, reverberation artifacts from the pleura, and absence of atelectasis^31^ (collapse) or consolidation (Fig. 3). Distinguishing between lung atelectasis^31^ (collapse) and consolidation is challenging on lung ultrasound, as the two frequently co-exist. For this preliminary study, consolidation and atelectasis were considered as one finding (I.e. collapse/consolidation). Collapse/consolidation was defined as an area of increased tissue density (tissue pattern) in the lung space that has the appearance of a solid organ, such as the liver (‘hepatization’). Other features used to report the presence of collapse/consolidation include air bronchograms (hyperechoic dots) and loss of lung volume, however these were not strictly required. Figure 3 demonstrates comparison LUS frames of an unhealthy (consolidation/collapse) vs healthy patient, from three different scan protocol regions. Out of the total 6 regions scanned during the examination, in our dataset, the RANT, RPL, and LPL regions contained imaging patterns consistent with lung consolidation/collapse. This resulted in the frames per LUS video varying anywhere from 50 to 300 frames, and for multiple scans (1-5 videos) per region. The distribution of unhealthy (consolidation/collapse) and healthy patients in the dataset and any coexisting lung pattern changes that were identified are shown in Table 1 and Fig. 2.

In Table 1, with respect to the first column (number of patients), the columns under the heading Ultrasound Classifications show the lung changes present, while the last 2 columns show the number of videos and frames associated with the patients of each row. The last row of the table describes the healthy patient’s dataset.

#### Dataset exclusion criteria

The image quality for unhealthy (consolidation/collapse patients) and healthy LUS videos/frames ruled out what patients would be included for both the training of the algorithm and the ground truth labelling done by the trained sonographers. Clinical experts, namely, an experienced sonographer and a certified medical doctor, labelled the dataset described in “Related works” section to provide the DL model with ground truth labels for training purposes. Each LUS frame was assigned a binary label indicating if it contained clinical signs of consolidation/collapse (Score 1) or not (Score 0). The images acquired from improper US probe placement or containing imaging artefacts that significantly impair the ability to identify consolidation/collapse imaging patterns were excluded from the consolidation/collapse dataset.

#### Criteria for clinical significance of algorithm performance

The ultrasound images were revised by a trained sonographer (MS) with the intent of providing a video-based evaluation of the sonographer’s confidence level in determining if a scanning region contains consolidation/collapse or not. Confidence is based on image quality, and many other contributing factors such as the ability to identify anatomical landmarks, and image artifacts present that hindered the conclusive identification of any given frame with either having consolidation/collapse present or not.

This criterion consisted in a 3-scoring system (I.e. Y, Y* and N) representing the level of confidence in identifying consolidation/collapse in each video. A label of “N” represents an inconclusive decision in determining both the scanning region and consolidation/collapse identification due to varying factors (such as improper probe placement, imaging artefacts obstructing ability to identify a given pathology by its associated imaging pattern, poor image quality, etc). “Y*” represents frames in the associated video having both a high confidence of frames containing consolidation/collapse as well as frames that are inconclusive in terms of anatomical landmarks identification. Finally, Y represents frames with a clear determination of consolidation/collapse being present and clear anatomical markers present.

The original dataset when considering the consolidation/collapse (along with any other associated pathology presented by identifying its associated imaging pattern), shows 11 patients or 40 videos (4910 frames) as shown in Table 1. the use of the exclusion and clinical criteria shown in Tables 2 and 3 led to a final consolidation/collapse training dataset of 9 patients or 23 videos (3827 frames) and is shown in “Video-based labelling strategies” section.

**Table 1:** Describes the pathology distribution among patients, after Dataset Review and exclusion (Fig. 2).

# of patients	Ultrasound classifications				# of videos	# of frames
	Consolidation/collapse	PE	APO	Interstitial syndrome		
3	$\checkmark$				9	1397
4	$\checkmark$	$\checkmark$			15	1682
3	$\checkmark$		$\checkmark$	$\checkmark$	17	2071
18	Healthy patients (no pathology present)				162	29167

Abbreviations: Acute Pulmonary Oedema (APO), Pleural Effusion (PE).

**Table 2:** The clinical criteria used to score LUS videos that have been evaluated by the algorithm beforehand. The labelling system determines a certain confidence of an experienced sonographer to identify the overall rating of a video based on the corresponding consolidation/collapse imaging pattern per frame using anatomical markers, image quality, and other possible obstructions for appropriate identification.

	Y	Y*	N
Obstructions to identification	Little to no ambiguity or obstructions to view	Possible causes: - artefacts overlappin - poor ultrasound probe placement	
Consolidation/collapse identified	Conclusive frames	Conclusive frames inconclusive frames	Inconclusive frames
Anatomical landmarks/scanning regions identified	Conclusive frames	Conclusive frames inconclusive frames	Inconclusive frames
Image quality	Good to high	Not optimal to normal	Not optimal/poor

### Frame-based labelling strategy

All frames of each unhealthy (consolidation/collapse patient) video were given a label of ’1’ (consolidation/collapse) or a label of ’0’ (no consolidation/collapse present). The frames labelled ’0’ were then excluded from the dataset. All frames of healthy patients were instead assigned a label of ’0’.

**Table 3:** Describes the consolidation/collapse dataset distribution before and after application of the data exclusion and clinical criteria.

	Frames per scanning region			
	RANT	RPL	LPL	Total
Original data	807	3065	1578	5450 Videos=4 Patients=11
Exclusion criteria	507	2825	1578	4910 Videos=35 Patients=10
Clinical criteria	507	2071	1249	3827Videos=23 Patients=9

Table 3 summarises the dataset initially into the total number of frames with consolidation/collapse demonstrated across the various scan protocol regions. It then further classifies this data into the number of frames used to train the algorithm after exclusion and clinical criteria have been applied. This exclusion process results in the ideal frames and image patterns of LUS patients with consolidation/collapse to be used to train the algorithm.

### Video-based labelling strategies

Besides the standard frame-based labelling approach described in “Video-based labelling strategies” section, video-based labelling strategies were also explored, as described “Pre-processing” section, to reduce labelling time.

While the training of the frame-based model is considered supervised learning, since frame-level predictions are evaluated using frame-level labels during training, the training of the video-based models is considered inaccurately supervised learning, a form of weakly supervised learning. This is because it is possible for the frame-level labels used for training to have errors^32^. Such errors result because each video-based labelling strategy involves using a subset of frames from each video to determine the single label that is given to all frames of the video.

#### All-or-nothing video-based labelling

**Figure 4:**
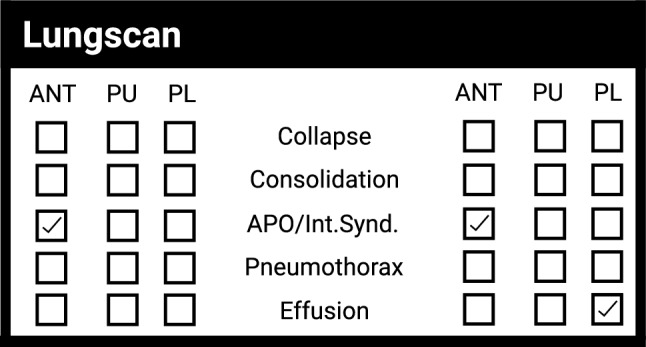
Example of the information provided by the medical report where each LUS scanning region consists of a video that has been checked marked if it contains a pathology.

For the all-or-nothing video-based approach, each patient was provided with iLungScan$^\mathrm{TM}$ (Heartweb Pty Ltd, ITeachU Ltd, ACN 146184812) reports from LUS experts, developed by the Ultrasound Education Group at the University of Melbourne and validated by other experts from the University of Melbourne and QUT. These reports state the severity of consolidation/collapse present (as well as other pathologies) in the six scanning positions and are marked with a checkmark as shown in Fig. 4.

Effectively, if one frame in a single video had consolidation/collapse present, all the remaining frames of the video would be incorrectly labelled as having consolidation/collapse. The all-or-nothing method was named after this limitation, whereby videos containing few frames with consolidation/collapse present produce more frames that are incorrectly labelled than are correctly labelled. The shortcoming is addressed by the sampled video-based labelling methods described in the following section.

#### Sampled binary and sampled quaternary video-based labelling

**Figure 5:**
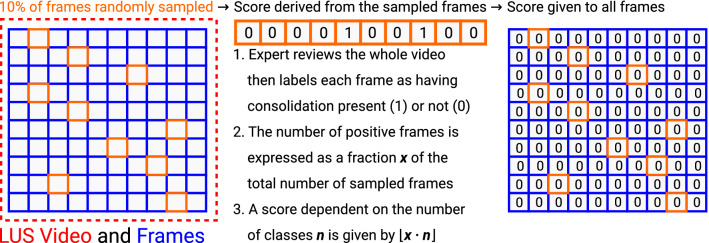
Illustrates the flowchart for the sampled binary and quaternary video-based labelling methods.

For the sampled binary and sampled quaternary labelling methods (Fig. 5), a label was assigned to a video and its associated frames, by comparing the number of consolidation/collapse frames as a percentage of the total number of frames for that video. For these sampled labelling methods, given an LUS video, 10% of its frames were randomly sampled rather than using periodic sampling to reduce systematic errors. In our case, the labels were from the frame-based labelling method (“Video-based labelling strategies” section) and were validated by the clinical experts (AW, MS) by assigning each frame a binary label of 0 (healthy) or 1 (unhealthy containing consolidation/collapse).

After the 10% sampling of frames from an LUS video, all frames of the video were then assigned the same label depending on the proportion of unhealthy (consolidation/collapse) sampled frames, i.e. frames containing signatures of pulmonary consolidation/collapse (Fig. 3). For the binary method, a label of 0 was given to all the frames in each video frames if less than half the sampled frames were unhealthy (consolidation/collapse). Otherwise, a label of 1 was given. For the quaternary method, on the other hand, if the proportion of unhealthy (consolidation/collapse) sampled frames was lower than 25%, a label of 0 was given. Labels of 1, 2 and 3 were assigned for a proportion less than 50%, a proportion less than 75% and for the remaining interval of 75%-100% inclusive, respectively.

### Pre-processing

An open-source DICOM processing package (Pydicom^33^) was used to extract the original pixel data from the compressed DICOM format. Next, the various overlays inside and outside the ultrasound sector, including text, watermarks, and trademarks from the ultrasound imaging system, were replaced with black background pixels. The final step included cropping the images from $960 \times 720$ pixels to a size of $806 \times 550$ pixels which contained the ultrasound sector to reduce the dataset size before being input into the DL model and only include the relevant information contained in the image.

### Cross-validation

As per^14,25^, the train-test splits were performed at the patient level during cross-validation, i.e. all the images of a given patient were either included in the training or in the test set. The patient split was performed by assigning a binary label of healthy or unhealthy with imaging patterns consistent with consolidation/collapse (i.e. 0 or 1, respectively) to each of the 28 patients using the medical reports described in “Related works” section. This could be done unambiguously because there were no patients for whom a mixture of healthy and unhealthy consolidation/collapse LUS videos was collected, as determined by the medical reports.

A Stratified 10-fold cross-validation was used where each test set contained frames from one consolidation/collapse patient, with the remaining video frames belonging to the healthy patients for which no imaging patterns resulting in any pathology was present. Since there was a total of 9 patients for which consolidation/collapse was present, one of the 10 folds had a test set containing only healthy patient video frames and was hence excluded from the final calculation. Note that for the sampled labelling methods of Section D2, the 10% sampling used to generate labels was performed once initially, rather than once per fold.

The trade-off between the quantity and quality of training examples is managed with the N/Y/Y* video categories from Section A2. Unlike the training examples from the videos labelled N, the Y* videos were deemed of high enough quality to be used for training in addition to the Y videos. However, since they did not possess the near-perfect inter-observer agreement of the Y videos, they were not considered of sufficient quality to be included in the test set to evaluate the algorithm performance. Hence ultimately the test set consisted solely of healthy videos and Y videos.

During the cross-validation process for training the algorithm, there was an equivalent number of frames between healthy and unhealthy consolidation/collapse frames per scanning region. Therefore, per training fold the scanning regions used for healthy and unhealthy consolidation/collapse frames was kept relatively the same to represent a training done on a balanced dataset. The distribution of the patients during training and testing of the model are presented in Table 4.

**Table 4:** Shows the distribution of the patients and their respective videos and frame during the training and testing of the model/algorithm.

Patient distribution after Exclusion and Clinical Criteria			
	Patients	Videos	Frames
Total	27	162	32994
Healthy	18	139	29167
Training	16/17	125–133	26125–27664
Testing	1/2	6–14	1248–2912
Consolidation/collapse	9	23	3827
Training	8/9	21–23	3486–3827
Testing	1	2	120–330

### Deep learning model

A DL architecture consisting of a CNN and Spatial Transformer Network (STN)^34^ was employed. Specifically, it used a Regularised Spatial Transformer Network (Reg-STN)^14^ to localise signatures of pulmonary consolidation/collapse. The Reg-STN uses ordinal labels (i.e. binary or quaternary labels in our case) as opposed to the explicit consolidation/collapse locations per frame^14^. It creates an image crop used by the CNN for feature extraction to ultimately produce a prediction^14^.

The algorithm was optimised using the same loss function as Roy S et al.^14^, who employed it for COVID-19 severity score estimation, an ordinal regression^35^ problem. This overall loss function, taking the form of a sum of terms, incorporated as one of its terms a soft ordinal regression (SORD)^36^ cross-entropy loss function to allow long-distance errors to be penalised harsher than low-distance errors^14^.

This model was selected since it is based on a unique/novel Reg-STN network, that has been proven to be able to localize and identify features in LUS images that were associated with COVID-19^14^.

### Training approach

Since the training and test sets were formed using stratified cross-validation, their class distributions reflected that of the whole dataset, with far healthier LUS frames than unhealthy consolidation/collapse frames. Hence, following^25^, a batch-level class balancing was implemented using the weighted random sampler from PyTorch^37,38^. As in^14^, the DL model was trained using an Adam optimiser with a learning rate decay of $1 \times 10^{-4}$, early stopping on the training loss, and online data augmentation ^14^. This training was run up to a maximum of 80 epochs, using a batch size of 32 and an initial learning rate of $1 \times 10^{-5}$. For the frame-based labelling, all-or-nothing video-based labelling, and binary video-based labelling the number of classes of the SORD loss function described in “Deep learning model” section was set to $n = 2$, while for the quaternary video-based labelling method $n = 4$ was used.

The network was trained on a single Nvidia Titan RTX GPU with 24 GB of memory installed on a workstation running Linux with 128GB of memory. The GPU workstation used an Intel i9-9820X CPU with 20 cores running at 3.30 GHz (Lambda Labs, San Francisco, CA, USA).

### Evaluations

#### Evaluation metrics

The models trained using both the frame-based labelling approach and the video-based labelling approaches produced frame-level predictions, which were evaluated against the frame-based binary ground truths as in^25^.

To evaluate the quaternary method in a manner comparable to the all-or-nothing and sampled binary methods, quaternary labels 0 and 1 were considered negatives (i.e. healthy) with the rest being considered positives (i.e. unhealthy or containing imaging patterns consistent with consolidation/collapse). That is, letting $\mathbf {a}$ denote the test set label and $\mathbf {p}$ denote the prediction, a linear projection from the $4 \times 4$ quaternary confusion matrix $\left[ Q_{\mathbf {ap}}\right]$ to the binary confusion matrix was defined by the equations

$$\begin{aligned} \mathrm {TP}= & {} Q_{2 2} + Q_{2 3} + Q_{3 2} + Q_{3 3} \\ \mathrm {FP}= & {} Q_{0 2} + Q_{0 3} + Q_{1 2} + Q_{1 3} \\ \mathrm {FN}= & {} Q_{2 0} + Q_{2 1} + Q_{3 0} + Q_{3 1} \\ \mathrm {TN}= & {} Q_{0 0} + Q_{0 1} + Q_{1 0} + Q_{1 1} \end{aligned}$$

which respectively define the true negatives $\mathrm {TN}$, false positives $\mathrm {FP}$, false negatives $\mathrm {FN}$, and true positives $\mathrm {TP}$ for the quaternary method. Note that $Q_{\mathbf {ap}} = 0$ when $a = 1$ or $a = 2$ since the frame-based ground truths are binary. This method allowed metrics such as accuracy, precision, recall, and F-score to be defined using binary formulae for the quaternary method and thereby compared to the same set of metrics applied to the frame-based method and the all-or-nothing and sampled binary video-based methods. A classification threshold of 0.5 was used to separate the positive and negative classes to evaluate these metrics and was not calibrated because the calibration would require us to reduce the size of our already small training and test set sizes to afford a validation set for threshold tuning.

Given this imbalance, the appropriate classification threshold-independent measure of skill is PR-AUC score, which does not account for true negatives and thereby exaggerate classifier performance, unlike ROC-AUC score^39^. Additionally, precision-recall curves^39^ were also used for evaluation. Each point on a precision-recall curve corresponds to a possible value for the classification threshold that separates negative and positive classes. This threshold is applied to the predicted score for the positive class, or the sum of the predicted scores for the positive classes (viz. 2 and 3) in the case of the quaternary method. Precision-recall curves are summarised by the Precision-Recall curve Area Under Curve score (PR-AUC), which corresponds to the average precision across the precision-recall curve.

#### Evaluation of statistical significance

To evaluate the significance of PR-AUC scores, the statistical procedure suggested by^40^, stratified bootstrapping, was used. Stratified bootstrapping involves drawing *n* positive samples and *m* negative samples from the dataset with replacement for each of $\mathcal {I}$ iterations to produce a distribution of $\mathcal {I}$ bootstrapped PR-AUC scores. Stratification is necessary because PR-AUC scores are sensitive to class imbalance^40^, with the *n* : *m* ratio giving the vertical centre point for a horizontal line, which is the precision-recall curve for a classifier with no skill.

Specifically, for each fold, given the test set predictions for a pair of labelling methods to be compared, a pair of PR-AUC scores was obtained, whose difference we refer to as the observed difference. To test if the two scores for each fold were significantly different from each other, and hence test the null hypothesis that the performances of the pair of labelling methods were insignificantly different, stratified bootstrapping with 10,000 iterations was employed. For each fold, two sets of bootstrapped PR-AUC scores, corresponding to a pair of labelling methods, were obtained and used to form the distribution of 10,000 PR-AUC score differences. Under the null hypothesis, the two sets of PR-AUC scores would have been sampled from the same distribution, so that the distribution of differences would have mean zero; the alternate hypothesis, on the other hand, is that the respective means of the two sets of bootstrapped scores are different. Hence, to form a distribution able to test the null hypothesis, the observed difference was subtracted from each of the 10,000 PR-AUC score differences to form a mean-shifted distribution of differences, from which a *p*-value was finally obtained by performing a *t*-test. Note that an identical *p*-value, for a fold and pair of labelling methods, could have been obtained by performing a paired-samples *t*-test on the two mean-shifted distributions of PR-AUC scores, as opposed to differences, corresponding to each labelling method.

Ultimately, recalling from “Cross-validation” section that one of the 10 folds was excluded, for each pair of labelling methods, 9 *p*-values corresponding to 9 folds were obtained. Each *p*-value indicated whether the pair of PR-AUC scores corresponding to the pair of labelling methods for a given fold were significantly different. A Bonferroni correction^41,42^ was used to correct for the multiple comparisons problem, whereby the chance of falsely rejecting the null hypothesis by chance alone increases with the number of repetitions of a family of hypothesis tests testing the same hypothesis. Therefore, a 5% chosen significance level was divided by 9 folds to yield a 0.56% Bonferroni-corrected significance level. Using this Bonferroni-corrected significance, if the null hypothesis were true, 5% of the tests performed are expected to have their null hypothesis rejected by chance alone. Hence, out of the 9 *p*-values obtained to compare labelling methods, it is sufficient that one of them (i.e. 11% of the *p*-values) is below the Bonferroni-corrected significance level of 0.56% to conclude that the labelling methods are significantly different.

#### Inter/intra-observer tests

To perform the inter/intra-observer test metrics, two independent (1 MD from Royal Melbourne, 1 clinically trained LUS sonographer) experts were tasked with performing clinical labelling of the original consolidation/collapse (as described in Table 1 of the original patient dataset). The labelling done by each expert comprised of a per frame binary scoring system where a score of 0 (no consolidation/collapse present) or a score of 1 (consolidation/collapse present) was assigned to all patients from Table 1. The scope of a given score of 0 includes frames that are conclusive for no imaging patterns associated with a given pathology being present, and inconclusive or indeterminate for imaging patterns associated with a pathology not being present and is further described (on a per video basis) in “Related works” section. The inter/intra-observer agreement was calculated using a percent agreement between the experts and the algorithm and a Cohen kappa score^43^ between the experts.

## Results

For the specific classification threshold of 0.5 that was used, with respect to accuracy, the frame-based method performed best (accuracy: 90.1%), with the video-based methods performing from best (accuracy: 88.7%) to worst (accuracy: 87.2%) in the following order: sampled quaternary, sampled binary, all-or-nothing binary (Table 5). However, given the class imbalance of the dataset (Tables 6, 7), accuracy reflects the ability of each method to produce true negatives. Indeed, the order of accuracies from the highest performing frame-based method to the lowest performing all-or-nothing binary method is identical to the order from highest to lowest of the percentage of true negatives produced (Table 7). Hence F1 score is a more suitable metric for evaluation. For the specific classification used, with respect to F1 score, the video-based methods (inaccurately supervised learning methods) outperformed the frame-based method (supervised learning method) with the sampled quaternary video-based method (F1 score: 67%) performing best and the frame-based method (F1 score: 55%) performing worst (Table 5).

When considering the class imbalance of the dataset, it is particularly important to calibrate the classification threshold^44^. Hence, other Table 5 metrics are less informative than the PR-AUC score, a classification threshold-independent metric. With respect to PR-AUC score, the Table 5 methods performed from best to worst in the same order as they performed for F1 score with the sampled quaternary video-based method performing best (PR-AUC score: 73%) and the frame-based method performing worst (PR-AUC score: 60%). Additionally, with respect to PR-AUC score, the sampled quaternary method outperformed the sampled binary method by 11% and the all-or-nothing binary method by 9% (Table 5).

**Table 5:** The mean/std test-set metrics for the 10-fold cross validation, for which videos labelled N (Section II.A2) were excluded. One of the 10 folds, which contained only healthy frames, was excluded from the results. Here Y* frames are frames Y* videos (Section II.A2).

	Mean/SD (%) with Y* frames excluded from the test set				
Method	PR-AUC	Recall	Precision	F1-score	Accuracy
Frame-based	60.08 ± 39.38	63.28 ± 36.62	53.01 ± 37.44	54.69 ± 38.02	90.18 ± 9.35
All-or-nothing binary video based	64.37 ± 39.32	69.18 ± 28.12	56.27 ± 37.86	59.10 ± 34.96	87.21 ± 17.07
Sampled binary video based	62.39 ± 40.09	69.22 ± 28.30	52.75 ± 36.37	55.92 ± 32.31	87.41 ± 14.97
Sampled quaternary video based	73.34 ± 30.37	83.67 ± 23.62	59.26 ± 28.14	66.78 ± 25.13	88.73 ± 15.87

**Figure 6:**
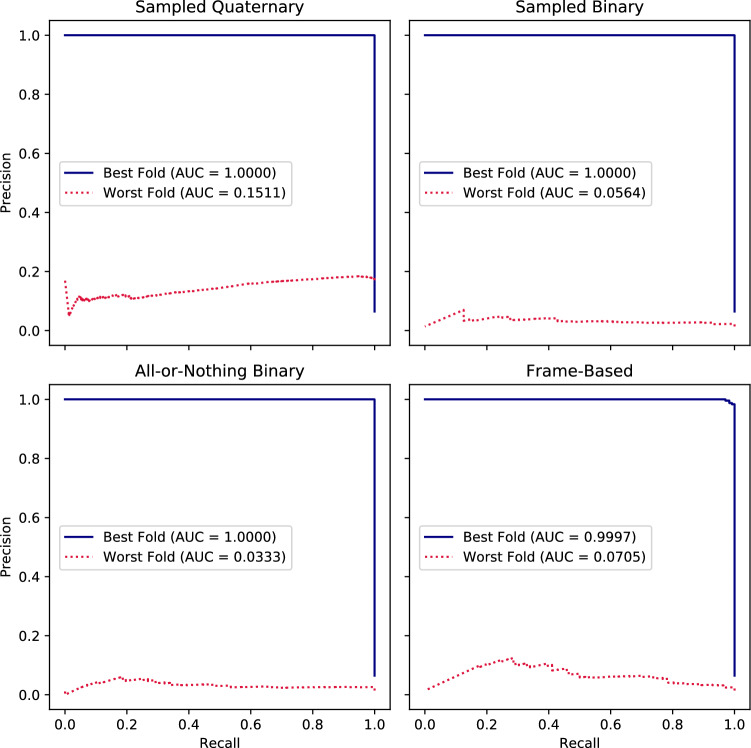
The best and worst fold test set precision-recall curves across the 10 folds for which videos labelled N (Section II.A2) were excluded from the training, and for which videos labelled Y* were excluded from the test set. These are given with their associated average precisions given by the Area Under Curve (AUC) scores, for models trained using the various labelling methods: the sampled quaternary and binary video-based methods, the all-or-nothing binary video-based method, and the frame-based method. One of the 10 folds, which contained only healthy frames, was excluded from the results.

The precision-recall curves of the best and worst folds are judged using PR-AUC score and given by Fig. 6. Recall that PR-AUC score is sensitive to the ratio between consolidation containing ground truth frames to the total number of the frames, which defines the precision-recall curve for a classifier with no skill. This ratio across folds (mean/std (%): 11.6 ± 8.1) has maximum 29.1% and minimum 1.4%.

As described in “Evaluation of statistical significance” section, a Bonferroni-corrected significance level of 0.56% was used to assess for each pair of labelling methods, the 9 *p*-values corresponding to 9 folds. In all cases, the percentages of folds for which the null hypothesis was rejected were significantly greater than the 5% rate expected due to chance if the scores corresponding to a pair of labelling methods were insignificantly different. More concretely, the sampled quaternary method was found to be significantly different compared to the sampled binary method (mean/std *p*: 0.12 ± 0.29) for 50% of folds, the all-or-nothing binary method (mean/std *p*: 0.17 ± 0.29) for 50% of folds, and the frame-based method (mean/std *p*: 0.17 ± 0.3) for 60% of folds. Additionally, the sampled binary method was found to be significantly different compared to the all-or-nothing binary method (mean/std *p*: 0.24 ± 0.26) for 30% of folds, and the frame-based method (mean/std *p*: 0.16 ± 0.27) for 60% of folds. Finally, the frame-based method was found to be significantly different from the all-or-nothing binary-based method (mean/std *p*: 0.19 ± 0.36) for 70% of folds.

The metrics here utilised for the analysis of the inter-observer agreement include the Cohen kappa and the percent agreement comparing the labels provided by two experts. In Table 6, in the columns under the heading Data after criteria, the percent agreement is used to show the agreement between two experts in labelling frames associated with imaging patterns from the consolidation/collapse pathology. This metric is calculated before and after the clinical criteria had been applied to demonstrate to what degree the experts agree on consolidation/collapse frames labelled Y (high image quality, conclusively identified imaging patterns associated with pathology, clear anatomical markers), labelled Y* (conclusively identified imaging patterns associated with pathology, exactly one of: unclear anatomical markers or lower image quality), and labelled N (e.g. lower image quality, inconclusively identified imaging patterns associated with pathology, unclear anatomical markers). The *Expert 1 / Algorithm* and *Expert 2 / Algorithm* rows show the percent agreements of each expert’s labels, and the classifier’s predictions for the test set, which includes a mixture of healthy and unhealthy consolidation/collapse patients for each of the labelling methods. Since the frames that did not satisfy the clinical criteria and were therefore labelled N were excluded from the algorithm training, these frames were also not considered in the percent agreement and Cohen kappa scores computations.

The quaternary method performs the best in terms of percent agreement with the worst performing method being the all-or-nothing video-based method (Table 6). The Cohen kappa/percent agreement for the (Y/Y*/N), (Y/Y*), and (N) cases between Expert 1 and Expert 2 are 0.537/0.805, 0.956/0.99, and 0.149/0.59 respectively. The percent agreement along with the other metrics (e.g. PR-AUC, accuracy) shows that the performance of the algorithm is at least on par if not at certain times slightly better than the trained experts after the application of the data exclusion criteria and the clinical criteria.

**Table 6:** Metrics for the inter-observer agreement analysis.

Comparison	% Agreement			
	Data after exclusion criteria			
	Frame-based	All-or-nothing video	Sampled binary video	Sampled quaternary video
Expert 1/algorithm	{Y and Y*}			
	(90.088)	(87.626)	(87.823)	(89.791)
	{Y}			
	(91.106)	(89.154)	(89.240)	(90.994)
Expert 2/algorithm	{Y and Y*}			
	(90.144)	(87.767)	(87.87.835)	(89.956)
	{Y}			
	(91.602)	(89.186)	(89.202)	(91.026)

(% agreement), {clinical criteria (unhealthy (consolidation/collapse)/healthy dataset) Y: Optimal, Y*: Good, N: Poor}.

**Table 7:** The mean/std test-set confusion matrix for the 10-fold cross validation test-set results with videos labelled N (Section II.A2) excluded. Positives correspond to frames from LUS videos that contain signatures of consolidations (and associated imaging patterns) while negatives correspond to frames that do not. One of the 10 folds, which contained only healthy frames, was excluded from the results. Here Y* frames are frames Y* videos (Section II.A2).

Method	Mean/SD (%) with Y* frames excluded from the test set			
	TP	FP	FN	TN
Frame-based	5.68 ± 4.66	5.93 ± 7.18	4.08 ± 5.52	84.31 ± 28.82
All-or-nothing binary video based	6.75 ± 3.90	9.74 ± 13.80	3.01 ± 3.46	80.50 ± 31.80
Sampled binary video based	6.73 ± 3.98	9.51 ± 11.43	3.03 ± 3.52	80.73 ± 30.94
Sampled quaternary video based	7.59 ± 4.08	8.16 ± 11.72	2.17 ± 3.42	82.08 ± 33.69

## Discussion

In this paper, we utilised an automated DL-based approach that identifies consolidation/collapses in LUS images to aid in the prognosis of late stages of COVID-19 induced pneumonia. Here we extend our previous work on pleural effusion pathology classification^25^ by proposing an improvement to its video-based method, namely, through our sampled quaternary video-based method. We have evaluated this quaternary method by comparing it with the frame-based method and video-based method of our previous pleural effusion work and with the sampled binary video-based approach.

There is a trade-off between the quantity and quality of training data: By increasing the quality of the dataset by excluding videos, we reduce our dataset size. This trade-off was managed by excluding N but not Y* video frames from the training set, preferring instead of excluding only Y* from the test set. This exclusion of Y* from the test set was done because, in order to evaluate how well the compared classifiers fare against the challenge of label noise, the test sets ground truths must be virtually free of it. For images that do not fall under the Y criteria (Section A2), the inter-observer agreement varies greatly as the identification of a given frame with an imaging pattern associated with consolidation/collapse gets more inconclusive due to a range of factors including varying image quality, location of key anatomical markers, artefacts hindering or obstructing the associated imaging pattern found with consolidation/collapse, and improper or poor placement of US probe resulting in unusable or highly questionable image. Since the difference between the N/Y*/Y categories is due to the image information becoming clearer and less ambiguous in the identification of the imaging patterns associated with consolidation/collapse, as expected, the agreement between the experts increases and becomes more consistent for the Y category, while it decreases for the categories Y* and N due to the inconsistency and complexity of identifying this pathology.

In this study, only 2 experts were compared in the interobserver study mainly due to the time and effort required to perform this task and the limited number of available trained experts in this field. In the future, additional experts could be involved to refine our current results. However, given our image classification into the three different categories, it is expected that the interobserver agreement that will be obtained for the category Y, where the image information is not ambiguous, would be comparable to our current result, whereas a possible decrease in interobserver performance could be expected for the categories Y* and N.

While it is hard to gauge the performance of the model with respect to false positives and false negatives, since those metrics are with respect to a specific classification threshold of 0.5, domain knowledge may be leveraged. In the case of false positives, for which the algorithm predicted the frame as containing consolidation/collapse when there was none, the algorithm sometimes labels possible artefacts that resemble consolidation/collapse or liver like features if located in the RPL scanning region. A possible approach to addressing this issue is by providing the anatomical information of the liver and accounting for that during the trainings.In the case of false negatives, for which the algorithm predicted the frame as being free from consolidation/collapse when it was instead present, the misclassified frames were sometimes drawn from LUS videos for which the consolidation/collapse was hidden beneath an inflated lung and was only visible when the patient exhaled. This limitation could be addressed similarly to the false positive case, by accounting for patient inhales/exhales during training. Alternately, patient breathing rhythm could be accounted for automatically by an algorithm that accounts for the temporal relationship between frames.

To evaluate our classifiers in a classification-threshold independent manner, the PR-AUC score was used in place of the more common ROC-AUC score. This is because the dataset employed had an imbalanced class distribution^39^. The imbalanced class problem was exacerbated by the fact that in the case of the quaternary labelling method, there are three decision thresholds to be calibrated as opposed to one. Moreover, for imbalanced datasets, as in this work where there is a majority of healthy patients, the TN, FP, FN,TP rates obtained were skewed which in turn resulted in a high discrepancy between the best and worst folds Precision-Recall curves (Fig. 6). In the future, these limitations will be solved using a larger dataset with a balanced number of pathologic and healthy cases.

The fact that the sampled quaternary method outperformed the sampled binary method with respect to the PR-AUC score is expected, and in fact this quaternary method performed best overall in this respect. This is because the quaternary approach performs a smoother transition between healthy and non-healthy classifications by providing 4 classes rather than 2. This increase in class granularity may be limiting the maximum ascent in training loss per training iteration due to the inherent error of video-based labelling in comparison to frame-based labelling. More concretely, the fact that the overall loss function used incorporated a SORD cross-entropy loss function as one of its terms allows long-distance errors to be penalised harsher than low-distance errors. This is beneficial because, just as some predicted severity scores are closer to the true severity score in^14^, some predicted sampled quaternary video-based method labels are more representative of the true number of frames with signatures of consolidation/collapse than others. This contrasts with cases where the classes are independent of each other.

In our findings, the inaccurately supervised learning of the video-based methods outperformed the supervised learning of the frame-based method with respect to PR-AUC score. This may be explained in terms of the bias-variance trade off, with the video-based methods shifting the trade off towards bias and the frame-based method shifting it towards variance. Indeed, for the video-based methods, a single label is being assigned to all frames of the video, so that the degrees of freedom the model has to overfit the noisy labelling data is reduced. Instead, a more uniform labelling noise is introduced across the frames of a video, which may be reducing the over-confidence of the video-based classifier has on any single frame. The quaternary method may be facilitating this reduction of over-confidence by preventing an overly high degree of noise from being injected uniformly across the labels of a video, as may be the case for the binary approaches. In this sense, our quaternary method is similar to label smoothing, a regularisation method that reduces over-confidence by smoothing labels and label noise, which been shown to be effective for datasets with incorrect labels present. Note, however, that the frame-based method still performed best in terms of accuracy, which may be due to the loss function used optimising error as opposed to a metric similar to PR-AUC score or F1 score that is more suitable for class imbalance scenarios.

While the sampled quaternary method outperformed the all-or-nothing binary method, there is a trade-off in terms of labelling effort. If medical reports are readily available, then the all-or-nothing method would not require additional labelling effort or clinical expertise. Hence, if the reduction in labelling time is of a higher priority than classification accuracy, then the all-or-nothing method may be preferred. The labelling effort of the sampled quaternary and sampled binary methods, on the other hand, are identical: this is because the labelling effort depends only on the percentage of frames sampled (10% in our case). Indeed, the sampled binary and quaternary methods are flexible in the sense that the trade-off between labelling effort and classification accuracy is easy to adjust: the higher the percentage of frames sampled, the higher the labelling effort.

A potential limitation of our findings relates to the fact that the dataset came from a single ultrasound system. Variability among manufacturers is expected. Therefore, the ability to generalize our findings to images acquired from other systems will need to be determined. The key limitation of our work was the reduced size of high-quality training examples. Future work could address this limitation through transfer learning, a more sophisticated approach to data augmentation than the online data augmentation that was used, or by applying our classifier to a larger and higher quality dataset. Each of these approaches mitigate overfitting, while the latter approach reduces the dataset label noise. Since overfitting and label noise are posited as the reason why our video-based approaches outperform the frame-based method, the suggested future work could provide evidence that our video-based approaches indeed mitigate label noise or overfitting in a manner similar to label smoothing. Additionally, due to the large number of healthy training examples and limitations on expert time, the Section A2 Y/Y*/N categorisation was not applied to the healthy examples. Other algorithms/models in the literature were trained to identify b-lines and pleural lining irregularities associated with COVID-19, but they were never tested on more or complex pathologies (PE, consolidation/collapse, atelectasis, interstitial syndrome, etc) and their associated imaging patterns. Furthermore, this model has the capability of performing both image classification and segmentation, which will be explored as part of our future work.

## Conclusion

Our work provides a tool for automatic consolidation/collapse identification of LUS video frames during point-of-care testing. Out of the labelling methods considered, the video-based methods were intended to reduce labelling effort while minimising the resulting loss of accuracy.

The video-based methods outperformed the frame-based method. This may be a result of overfitting due to the variance of our small dataset size or label noise. Specifically, our video-based methods may be more robust to label noise and variance than the frame-based method. That is, in the bias-variance trade-off the frame-based method shifts the trade-off towards variance while the video-based method shifts the trade-off towards bias. It is expected that if the classifier was run on a larger dataset of higher quality, the frame-based method would outperform the video-based methods. However, this must be confirmed through future work.

The best performing method was the sampled quaternary method, which employed a novel training approach using four classes, and performed better than the medical report based all-or-nothing method of^25^. However, if medical reports corresponding to LUS videos are readily available, then the all-or-nothing method may be preferred for scenarios where a reduction in labelling time is prioritised higher than classification accuracy.

## Data Availability

The datasets used and/or analysed during the current study are available from the corresponding author(s) on reasonable request.
